# Enhancing Reporting Quality Using the Preferred Reporting Items for Systematic Review and Meta-Analysis 2020 in Systematic Reviews of Emergency Medicine Journals: A Cross-Sectional Study

**DOI:** 10.7759/cureus.78255

**Published:** 2025-01-30

**Authors:** Chiaki Suda, Norio Yamamoto, Takahiro Tsuge, Minoru Hayashi, Kosuke Suzuki, Yasuhisa Ikuta, Masahiro Banno

**Affiliations:** 1 Department of Public Health, Gunma University Graduate School of Medicine, Maebashi, JPN; 2 Department of Orthopedic Surgery, Minato Medical Coop-Kyoritsu General Hospital, Nagoya, JPN; 3 Department of systematic reviewers, Systematic Review Workshop Peer Support Group (SRWS-PSG), Osaka, JPN; 4 Department of Epidemiology, Graduate School of Medicine, Dentistry and Pharmaceutical Sciences, Okayama University, Okayama, JPN; 5 Department of Systematic Reviewers, Systematic Review Workshop Peer Support Group (SRWS-PSG), Osaka, JPN; 6 Department of Emergency Medicine, Fukui Prefectural Hospital, Fukui, JPN; 7 Department of Rehabilitation, Yamagata Saisei Hospital, Yamagata, JPN; 8 Division of Neonatology, Center for Maternal-Fetal, Neonatal and Reproductive Medicine, National Center for Child Health and Development, Tokyo, JPN; 9 Department of Psychiatry, Seichiryo Hospital, Nagoya, JPN

**Keywords:** emergency medicine, meta-epidemiological study, prisma 2020, prisma guidelines, reporting quality

## Abstract

Background: Systematic reviews (SRs) with thorough reporting and rigorous methodology lead to less biased outcomes. The Priority Reporting Items for Systematic Reviews and Meta-Analyses (PRISMA) statement was developed to enhance SRs and meta-analysis reporting. While it was updated to PRISMA 2020, the impact on emergency medicine remains unexplored. Therefore, we aimed to investigate whether using PRISMA 2020 improves the reporting quality of SRs in the emergency medicine field.

Methods: This study is a cross-sectional meta-epidemiological analysis of SRs published in emergency medicine journals between 2021 and 2023. We selected SRs with pairwise meta-analyses of health interventions included in MEDLINE. We evaluated adherence to PRISMA 2020 items with and without the use of the PRISMA 2020 statement.

Results: A total of 695 articles were analyzed, ultimately including 31 that used PRISMA 2020 and 100 that did not. Adherence rates to PRISMA 2020 items were higher in papers using PRISMA 2020 (925/1270, 72.8%, odds ratio: 1.24, 95% confidence interval: 1.08-1.43) than in papers not using it (2758/4034, 68.4%). No SRs met all of the PRISMA 2020 criteria. Adherence to PRISMA 2020 for Abstracts was slightly higher in the group that used the PRISMA 2020 (182/372, 48.9%, odds ratio: 1.17, 95% confidence interval: 0.92-1.47), compared to those that had not (541/1200, 45.1%). Adherence was highest in the introduction and lowest in the methods section. Agreement between the first author and other reviewers’ ratings averaged 89.6% (4815/5371).

Conclusion: Implementing PRISMA 2020 significantly improved the reporting quality of SRs in emergency medicine-related journals. Declaring the use of PRISMA 2020 is insufficient, and researchers must strictly adhere to each item.

Registration: The study protocol was registered in the Open Science Framework on September 22, 2023 (https://doi.org/10.17605/OSF.IO/DQ5W6).

## Introduction

Thorough reporting and rigorous methodology in systematic reviews (SRs) lead to less biased recommendations and decisions [1]. Emphasis is placed on reporting and methodology quality to ensure clarity and transparency regarding research implementation procedures [2,3]. In 2009, the Priority Reporting Items for Systematic Reviews and Meta-Analyses (PRISMA) statement was issued to help improve SR and meta-analysis reporting [4]. In 2020, the statement was updated as the PRISMA 2020 statement and provides updated reporting guidance for SRs that reflects advances in methods to identify, select, appraise, and synthesize studies [5,6]. The PRISMA 2020 statement improves areas affecting reporting quality by detailing protocol descriptions, recommending that all relevant databases, registries, and websites be searched, reporting excluded studies, stating the number of individuals screened and whether they worked independently, detailing the synthesis of results, and reporting conflicts of interest [7].

A prior study examining the PRISMA 2020 statement items found that SRs in radiology and inventions were not adequately reported [8,9]. The SRs of biomedical research, radiomics, rehabilitation, orthopedics, and surgery were also of suboptimal quality, according to PRISMA 2009 [10-19]. In emergency medicine journals, the SR reporting quality is still unclear, as there is no prior research on using the PRISMA 2020 statement.

In this study, we aimed to assess the impact of the PRISMA 2020 statement on the reporting standards of SRs in emergency medicine journals. The protocol of this study was previously posted to the osf.io preprint server on September 21, 2023 (https://osf.io/dq5w6/).

## Materials and methods

Study design and protocol

The protocol of this cross-sectional meta-epidemiological study is registered at osf.io (https://osf.io/dq5w6/). This study adheres to the reporting guidelines for meta-epidemiological studies [20]. We used publicly available data. Ethical approval and patient consent were not required.

Study selection and search

We selected SRs with pairwise meta-analyses on the effects of health interventions published in emergency medicine journals in 2021, 2022, and 2023 (Appendices: Figure 4). In accordance with our search criteria, no articles published before 2020 were included in the search results. We followed the definition of SR by Moher et al. [4,21] and PRISMA for SR protocols (PRISMA-P) [2]. We included only SRs written in English. Individual patient data analyses, network meta-analyses, and scoping reviews were excluded. Emergency medicine journals are defined based on the Journal Citation Report (https://jcr.clarivate.com/jcr/home). Of 47 emergency medicine journals defined based on the “emergency” category of the Journal Citation Report, we excluded 10 that were not published in MEDLINE (PubMed), resulting in 37 journals (Appendices: Table 5). We defined publication dates in 2021, 2022, and 2023 as electronic pre-publications or traditional journal publication dates. We searched for interventional SRs with pairwise meta-analyses published in emergency medicine journals in 2021, 2022, and 2023 using MEDLINE (PubMed) [22]. Our search strategies are outlined in Table 6 (Appendices). We defined exposure as the use of the PRISMA 2020 statement if the SR contained the term “PRISMA 2020,” cited “PRISMA 2020,” or used the PRISMA 2020 flow diagram. The control group consisted of SRs that did not use the PRISMA 2020 statement, including those related to PRISMA, such as the PRISMA 2009 statement. The outcome measure was adherence to the PRISMA 2020 items.

Screening

First, we randomly sampled 200 articles from the potentially included articles using a random number generator in Microsoft Excel (Microsoft Corporation, Redmond, WA, USA). We assigned random numbers to each data point using the RAND function and avoided duplicates by using the RANK function. These ranked random numbers were then used to extract 200 data points based on their ranks. Second, two of five reviewers (CS, TT, MH, KS, and YI) independently screened titles, abstracts, and full texts to identify articles that used or did not use the PRISMA 2020 statement and interventional SRs with pairwise meta-analyses. Random sampling was planned until a target of 100 articles was obtained for each group. Random sampling was adopted because 695 review subjects were identified, and resource constraints were taken into consideration. Ultimately, all 695 articles were screened. Random sampling was repeated until the SRs of one group reached 100. The number of SRs included in each group was capped at 100. Only 31 articles that used PRISMA 2020 were identified, so we included all of them in the group to use PRISMA 2020. Disagreements between reviewers were resolved by discussion; if this was not possible, a third reviewer arbitrated the disagreements (NY or MB).

Data extraction

Two of the five reviewers (CS, TT, MH, KS, and YI) independently extracted data from the included studies using a standardized data collection form. We collected the following epidemiological and reporting characteristics: country or region of the first author, number of authors, number of included studies, protocol registration, funding support, conflicts of interest, attachment of the PRISMA 2020 checklist, and use of the PRISMA 2009 statement. Two of the five reviewers independently evaluated adherence to the PRISMA 2020 items and PRISMA 2020 for Abstracts for the included articles. In the PRISMA 2020 items and PRISMA 2020 for Abstracts, we assessed adherence as “Yes” or “No.” Any disagreements were resolved through discussion; however, a third reviewer (NY or MB) functioned as an arbiter if necessary. For the group not to use the PRISMA 2020, we examined the quality of reporting by publication date. The base date was March 29, 2022 (1 year after PRISMA 2020 was published), and the pre- and post-PRISMA 2020 adherence rates were compared.

Statistical analysis

We summarized the data as the frequency for categorical data or median and interquartile range (IQR) for continuous data. We compared adherence to the PRISMA 2020 items and PRISMA 2020 for Abstracts items with or without using the PRISMA 2020 statement and calculated odds ratios (ORs) and 95% confidence intervals (CIs). We based the adherence rate to each item on a standard of at least 80% and not less than 50% [13]. The adherence rate was defined as the percentage of items for which multiple reviewers assessed the description as sufficient. We compared the assessments by the first author (CS) with other reviewers’ (TT, MH, KS, and YI) assessments of each item on the PRISMA 2020 checklist. We described the results as the proportion of agreement between the first author and other reviewers’ assessments for each item in the PRISMA 2020 statement. Statistical significance was assessed by 95% CIs. All analyses were performed using R Statistical Software (version 4.3.3; R Foundation for Statistical Computing, Vienna, Austria).

Differences between the protocol and article

Although we planned to examine 100 SRs using PRISMA 2020, only 31 SRs met the criteria, resulting in a small sample.

## Results

Selection process

The study selection process is summarized in the PRISMA 2020 flowchart (Figure 4). We identified 695 articles after performing a search on September 26, 2023. Since fewer articles than expected were found, so we screened all of them. After screening the titles and abstracts of the selected articles, 159 relevant articles were identified. We screened 159 full texts and included 31 articles that used the PRISMA 2020 statement and 107 that did not. We excluded 21 articles (Appendices: Table 7) that did not meet our criteria. In total, 138 articles meeting the eligibility criteria were adopted. However, seven articles were subsequently excluded because they exceeded the upper limit of 100 articles in the group not using the PRISMA 2020. At the protocol stage, the plan was to randomly select 100 pertinent references for each group. Because there were fewer than 100 references for the group to use the PRISMA 2020, all references were included. A random sampling was conducted, and 31 articles were included in the group to use the PRISMA 2020, and 100 were included in the group not to use the PRISMA 2020.

Characteristics of included articles

The epidemiological characteristics of the SRs for each group are summarized in Table 1.

**Table 1:** Epidemiological characteristics of systematic reviews in the groups to use PRISMA 2020 and not to use PRISMA 2020 PRISMA: Preferred Reporting Items for Systematic Reviews and Meta-Analyses

		The group to use the PRISMA2020 (n = 31)	The group not to use the PRISMA2020 (n = 100)
Country region of the first author	Asia	7 (23%)	47 (47%)
	Europe	5 (16%)	32 (32%)
	North America	8 (26%)	15 (15%)
	Oceania	6 (19%)	3 (3%)
	Middle East	3 (10%)	2 (2%)
	Central and South America	2 (6%)	1 (1%)
Number of authors	< 5	7 (23%)	37 (37%)
	≥ 5	24 (77%)	63 (63%)
Number of included studies	< 10	17 (55%)	51 (51%)
	≥ 10	14 (45%)	49 (49%)
Protocol Registration	Yes	19 (61%)	45 (45%)
	No	12 (39%)	55 (55%)
Funding support	Yes	7 (23%)	23 (23%)
	No	18 (58%)	54 (54%)
	No Information	6 (19%)	23 (23%)
Conflict of interest	Yes	1 (3%)	5 (5%)
	No	30 (97%)	91 (91%)
	No Information	0 (0%)	4 (4%)
Attachment of the PRISMA 2020 checklist	Yes	9 (29%)	-
Use of the PRISMA 2009	Yes	-	78 (78%)

North America had the most publications that use the PRISMA 2020 statement. Asia had the most publications that did not use the PRISMA 2020 statement. Specifically, China had the most publications, with 4 articles that used the PRISMA 2020 statement and 32 that did not. The median number of authors who used the PRISMA 2020 statement was 6.0 (IQR, 5.0-8.0) and 5.0 (IQR, 4.0-7.0) for articles that did not. The median number of included articles was 9.0 (IQR, 6.0-14.0) in both groups. Nineteen (61.3%) protocol registrations were identified for articles that used the PRISMA 2020 statement and 45 (45.0%) for those that did not. Among the articles that did not use the PRISMA 2020 statement, 78 (78%) used the PRISMA 2009 statement.

Evaluation of adherence to the PRISMA 2020 items

The mean overall adherence among our sample was 69.4% (3683/5304): 72.8% (925/1270, OR: 1.24, 95% CI: 1.08-1.43) in the group to use the PRISMA 2020 and 68.4% (2758/4034) in the group not to use the PRISMA 2020. Adherence rates were significantly higher in the group using PRISMA 2020 compared to the group not using PRISMA 2020. The mean adherence varied across items (Table 2, Appendices: Figure 5).

**Table 2:** Adherence with each item of the PRISMA 2020 *Excluding those not applicable to the item PRISMA: Preferred Reporting Items for Systematic Reviews and Meta-Analyses

Section and topic	Checklist item	Item#	The group to use the PRISMA 2020 (n=31)	The group not to use the PRISMA 2020 (n=100)	Odds ratio (95%CI)
Title					
Title	Identify the report as a systematic review.	1	27 (87%)	73 (73%)	2.50 (0.80-7.80)
Abstract					
Abstract	See the PRISMA 2020 for Abstracts checklist (Table 3).	2			
Introduction					
Rationale	Rationale 3 Describe the rationale for the review in the context of existing knowledge.	3	31 (100%)	100 (100%)	0.31 (0.01-16.12)
Objectives	Provide an explicit statement of the objective(s) or question(s) the review addresses.	4	30 (97%)	100 (100%)	0.10 (0.00-2.55)
Methods					
Eligibility criteria	Specify the inclusion and exclusion criteria for the review and how studies were grouped for the syntheses.	5	26 (84%)	78 (78%)	1.47 (0.50-4.27)
Information sources	Specify all databases, registers, websites, organizations, reference lists, and other sources searched or consulted to identify studies. Specify the date when each source was last searched or consulted.	6	22 (71%)	61 (61%)	1.56 (0.65-3.74)
Search strategy	Present the full search strategies for all databases, registers, and websites, including any filters and limits used.	7	16 (52%)	51 (51%)	1.02 (0.46-2.29)
Selection process	Specify the methods used to decide whether a study met the inclusion criteria of the review, including how many reviewers screened each record and each report retrieved, whether they worked independently, and if applicable, details of automation tools used in the process.	8	24 (77%)	72 (72%)	1.33 (0.52-3.44)
Data collection process	Specify the methods used to collect data from reports, including how many reviewers collected data from each report, whether they worked independently, any processes for obtaining or confirming data from study investigators, and if applicable, details of automation tools used in the process.	9	18 (58%)	69 (69%)	0.62 (0.27-1.43)
Data items	List and define all outcomes for which data were sought. Specify whether all results that were compatible with each outcome domain in each study were sought (e.g., for all measures, time points, analyses), and if not, the methods used to decide which results to collect.	10a	18 (58%)	76 (76%)	0.44 (0.19-1.02)
	List and define all other variables for which data were sought (e.g., participant and intervention characteristics, funding sources). Describe any assumptions made about any missing or unclear information.	10b	19 (61%)	76 (76%)	0.50 (0.21-1.18)
Study risk of bias assessment	Specify the methods used to assess the risk of bias in the included studies, including details of the tool(s) used, how many reviewers assessed each study and whether they worked independently, and if applicable, details of automation tools used in the process.	11	25 (81%)	68 (68%)	1.96 (0.73-5.25)
Effect measures	Specify for each outcome the effect measure(s) (e.g., risk ratio, mean difference) used in the synthesis or presentation of results.	12	28 (90%)	94 (94%)	0.60 (0.14-2.54)
Synthesis methods	Describe the processes used to decide which studies were eligible for each synthesis (e.g., tabulating the study intervention characteristics and comparing against the planned groups for each synthesis (item #5)).	13a	26 (84%)	78 (78%)	1.47 (0.50-4.27)
	Describe any methods required to prepare the data for presentation or synthesis, such as handling of missing summary statistics, or data conversions.	13b	6 (19%)	14 (14%)	1.47 (0.51-4.23)
	Describe any methods used to tabulate or visually display the results of individual studies and syntheses.	13c	14 (45%)	27 (27%)	2.23 (0.97-5.13)
	Describe any methods used to synthesize results and provide a rationale for the choice(s). If meta-analysis was performed, describe the model(s), method(s) to identify the presence and extent of statistical heterogeneity, and software package(s) used.	13d	22 (71%)	85 (85%)	0.43 (0.17-1.12)
	Describe any methods used to explore possible causes of heterogeneity among study results (e.g. subgroup analysis, metaregression).	13e	21 (68%)	61 (61%)	1.34 (0.57-3.15)
	Describe any sensitivity analyses conducted to assess the robustness of the synthesized results.	13f	17 (55%)	49 (49%)	1.26 (0.56-2.84)
Reporting bias assessment	Describe any methods used to assess the risk of bias due to missing results in a synthesis (arising from reporting biases).	14	24 (77%)	53 (53%)	3.04 (1.20-7.70)
Certainty assessment	Describe any methods used to assess certainty (or confidence) in the body of evidence for an outcome.	15	15 (48%)	35 (35%)	1.74 (0.77-3.94)
Results					
Study selection	Describe the results of the search and selection process, from the number of records identified in the search to the number of studies included in the review, ideally using a flow diagram.	16a	31 (100%)	97 (97%)	2.26 (0.11-44.99)
	Cite studies that might appear to meet the inclusion criteria, but which were excluded, and explain why they were excluded.	16b	13 (42%)	34 (34%)	1.40 (0.61-3.20)
Study characteristics	Cite each included study and present its characteristics.	17	30 (97%)	98 (98%)	0.61 (0.05-6.99)
Risk of bias in studies	Present assessments of risk of bias for each included study.	18	27 (87%)	75 (75%)	2.25 (0.72-7.06)
Results of individual studies	For all outcomes, present, for each study: (a) summary statistics for each group (where appropriate) and (b) an effect estimate and its precision (e.g. confidence/credible interval), ideally using structured tables or plots.	19	31 (100%)	85 (85%)	11.42 (0.66-196.59)
Results of syntheses	For each synthesis, briefly summarize the characteristics and risk of bias among contributing studies.	20a	13 (42%)	31 (31%)	1.61 (0.70-3.69)
	Present results of all statistical syntheses conducted. If meta-analysis was done, present for each the summary estimate and its precision (e.g. confidence/credible interval) and measures of statistical heterogeneity. If comparing groups, describe the direction of the effect.	20b	31 (100%)	98 (98%)	1.60 (0.07-34.20)
	Present results of all investigations of possible causes of heterogeneity among study results.	20c	25 (81%)	63 (63%)	2.45 (0.92-6.51)
	Present results of all sensitivity analyses conducted to assess the robustness of the synthesized results.	20d	19 (61%)	46 (46%)	1.86 (0.82-4.23)
Reporting biases	Present assessments of risk of bias due to missing results (arising from reporting biases) for each synthesis assessed.	21	20 (67%)*	50 (56%)*	1.6(0.67-3.80)
Certainty of evidence	Present assessments of certainty (or confidence) in the body of evidence for each outcome assessed.	22	14 (45%)	30 (30%)	1.92 (0.84-4.39)
Discussion					
Discussion	Provide a general interpretation of the results in the context of other evidence.	23a	31 (100%)	100 (100%)	0.31 (0.01-16.12)
	Discuss any limitations of the evidence included in the review.	23b	31 (100%)	99 (99%)	0.95 (0.04-23.91)
	Discuss any limitations of the review processes used.	23c	26 (84%)	81 (81%)	1.22 (0.41-3.59)
	Discuss the implications of the results for practice, policy, and future research.	23d	31 (100%)	92 (92%)	5.79 (0.32-103.21)
Other information					
Registration and protocol	Provide registration information for the review, including the register name and registration number, or state that the review was not registered.	24a	23 (74%)	56 (56%)	2.26 (0.92-5.53)
	Indicate where the review protocol can be accessed, or state that a protocol was not prepared.	24b	10 (32%)	48 (48%)	0.52 (0.22-1.21)
	Describe and explain any amendments to information provided at registration or in the protocol.	24c	19 (61%)	41 (93%)*	0.12 (0.03-0.46)
Support	Describe sources of financial or non-financial support for the review, and the role of the funders or sponsors in the review.	25	24 (77%)	77 (77%)	1.02 (0.39-2.68)
Competing interests	Declare any competing interests of review authors.	26	31 (100%)	97 (97%)	2.26 (0.11-44.99)
Availability of data, code, and other materials	Report which of the following are publicly available and where they can be found: template data collection forms; data extracted from included studies; data used for all analyses; analytic code; any other materials used in the review.	27	16 (52%)	40 (40%)	1.60 (0.71-3.60)

Of the 41 items (excluding item #2) in the PRISMA 2020 statement, the percentage of items that were reported in more than 80% of the literature was 43.9% (18/41) in the group to use the PRISMA 2020 and 34.1% (14/41) in the group not to use the PRISMA 2020. Of the same 41 items, the percentage reported in less than 50% of the literature was 14.6% (6/41) in the group to use the PRISMA 2020 and 22.0% (9/41) in the group not to use the PRISMA 2020. None of the included reviews fulfilled all the criteria of the PRISMA 2020 Statement. After assessing each item in the PRISMA 2020 statement, the reporting quality of item #14 (Reporting bias assessment; Describe any methods used to assess the risk of bias due to missing results in a synthesis (arising from reporting biases)) improved (OR: 3.04, 95% CI: 1.20-7.70) among articles in the group to use the PRISMA 2020 compared with those in the group not to use the PRISMA 2020. However, no significant differences were observed in the other items. The introduction section had the highest adherence rate (99.6%; 261/262), and the methods section had the lowest (62.3%; 1388/2227).

The mean overall adherence to the PRISMA 2020 for Abstracts was 46.0% (723/1572): 48.9% (182/372, OR: 1.17, 95% CI: 0.92-1.47) in the group to use the PRISMA 2020 and 45.1% (541/1200) in the group not to use the PRISMA 2020. Of the 12 items in the PRISMA 2020 for Abstracts, the percentage of items that were reported in more than 80% of the literature was 33.3% (4/12) in the group to use the PRISMA 2020 and 25.0% (3/12) in the group not to use the PRISMA 2020 (Table 3, Appendices: Figure 6). Of the same 12 items, the percentage reported in less than 50% of the literature was 58.3% (7/12) in both groups.

**Table 3:** Adherence with each item of the PRISMA 2020 for Abstracts PRISMA: Preferred Reporting Items for Systematic Reviews and Meta-Analyses

Section and topic	Checklist item	Item #	The group to use the PRISMA 2020 （n = 31）	The group not to use the PRISMA 2020 （n = 100）	Odds ratio (95%CI)
Title	Title	1	26 (84%)	77 (77%)	1.55 (0.54-4.50)
Background	Objectives	2	30 (97%)	97 (97%)	0.93 (0.09-9.25)
Methods	Eligibility criteria	3	1 (3%)	3 (3%)	1.08 (0.11-10.75)
	Information sources	4	13 (42%)	23 (23%)	2.42 (1.03-5.67)
	Risk of bias	5	9 (29%)	23 (23%)	1.37 (0.55-3.38)
	Synthesis of results	6	12 (39%)	34 (34%)	1.23 (0.53-2.82)
Results	Included studies	7	19 (61%)	73 (73%)	0.59 (0.25-1.37)
	Synthesis of results	8	27 (87%)	85 (85%)	1.19 (0.36-3.90)
Discussion	Limitations of evidence	9	12 (39%)	23 (23%)	2.11 (0.89-5.00)
	Interpretation	10	30 (97%)	99 (99%)	0.30 (0.02-4.99)
Other	Funding	11	0 (0%)	0 (0%)	3.19 (0.06-164.11)
	Registration	12	3 (10%)	4 (4%)	2.57 (0.54-12.18)

After assessing each item in the PRISMA 2020 for Abstracts, the reported quality in item #4 (Information sources; Specify the information sources (e.g. databases, registers) used to identify studies and the date when each was last searched.) improved (OR: 2.42, 95% CI: 1.03-5.67) among articles in the group to use the PRISMA 2020 compared with those in the group not to use the PRISMA 2020. However, no significant differences were observed in the other items.

**Figure 1:**
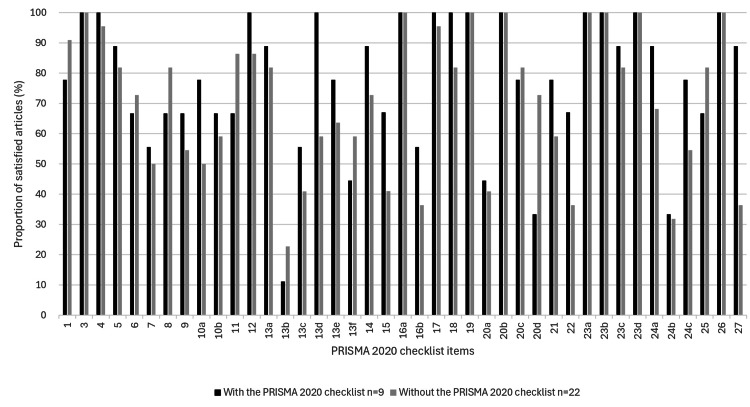
Bar chart of the adherence rate to each item of the PRISMA 2020 with or without the checklist Black bars indicate the use of the PRISMA 2020 checklist. Gray bars indicate no use of the PRISMA 2020 checklist. PRISMA: Preferred Reporting Items for Systematic Reviews and Meta-Analyses The image was created by the first author, Dr. Suda.

Among the group to use the PRISMA 2020, adherence rates for each item are shown based on whether the PRISMA 2020 checklist was attached (Table 4, Figure 1). Checklist use significantly improved adherence rates only for item #27 (Availability of data, code, and other materials; Report which of the following are publicly available and where they can be found: template data collection forms; data extracted from included studies; data used for all analyses; analytic code; any other materials used in the review.)

**Table 4:** Adherence with each item of the PRISMA 2020 with or without a checklist *Excluding those not applicable to the item PRISMA: Preferred Reporting Items for Systematic Reviews and Meta-Analyses

Section and topic	Checklist item	Item#	With the PRISMA 2020 checklist (n=9)	Without the PRISMA 2020 checklist (n=22)	Odds Ratio（95%CI）
Title					
Title	Identify the report as a systematic review.	1	7 (78%)	20 (91%)	0.35 (0.04-2.98)
Abstract					
Abstract	See the PRISMA 2020 for Abstracts checklist (Table 3).	2			
Introduction					
Rationale	Rationale 3 Describe the rationale for the review in the context of existing knowledge.	3	9 (100%)	22 (100%)	0.42 (0.01-22.96)
Objectives	Provide an explicit statement of the objective(s) or question(s) the review addresses.	4	9 (100%)	21 (95%)	1.33 (0.05-35.74)
Methods					
Eligibility criteria	Specify the inclusion and exclusion criteria for the review and how studies were grouped for the syntheses.	5	8 (89%)	18 (82%)	1.78 (0.17-18.53)
Information sources	Specify all databases, registers, websites, organizations, reference lists, and other sources searched or consulted to identify studies. Specify the date when each source was last searched or consulted.	6	6 (67%)	16 (73%)	0.75 (0.14-4.00)
Search strategy	Present the full search strategies for all databases, registers, and websites, including any filters and limits used.	7	5 (56%)	11 (50%)	1.25 (0.26-5.94)
Selection process	Specify the methods used to decide whether a study met the inclusion criteria of the review, including how many reviewers screened each record and each report retrieved, whether they worked independently, and if applicable, details of automation tools used in the process.	8	6 (67%)	18 (82%)	0.44 (0.08-2.58)
Data collection process	Specify the methods used to collect data from reports, including how many reviewers collected data from each report, whether they worked independently, any processes for obtaining or confirming data from study investigators, and if applicable, details of automation tools used in the process.	9	6 (67%)	12 (55%)	1.67 (0.33-8.42)
Data items	List and define all outcomes for which data were sought. Specify whether all results that were compatible with each outcome domain in each study were sought (e.g., for all measures, time points, analyses), and if not, the methods used to decide which results to collect.	10a	7 (78%)	11 (50%)	3.50 (0.59-20.75)
	List and define all other variables for which data were sought (e.g., participant and intervention characteristics, funding sources). Describe any assumptions made about any missing or unclear information.	10b	6 (67%)	13 (59%)	1.38 (0.27-7.04)
Study risk of bias assessment	Specify the methods used to assess the risk of bias in the included studies, including details of the tool(s) used, how many reviewers assessed each study and whether they worked independently, and if applicable, details of automation tools used in the process.	11	6 (67%)	19 (86%)	0.32 (0.05-2.00)
Effect measures	Specify for each outcome the effect measure(s) (e.g., risk ratio, mean difference) used in the synthesis or presentation of results.	12	9 (100%)	19 (86%)	3.41 (0.16-73.28)
Synthesis methods	Describe the processes used to decide which studies were eligible for each synthesis (e.g., tabulating the study intervention characteristics and comparing against the planned groups for each synthesis (item #5)).	13a	8 (89%)	18 (82%)	1.78 (0.17-18.53)
	Describe any methods required to prepare the data for presentation or synthesis, such as handling of missing summary statistics, or data conversions.	13b	1 (11%)	5 (23%)	0.43 (0.04-4.26)
	Describe any methods used to tabulate or visually display the results of individual studies and syntheses.	13c	5 (56%)	9 (41%)	1.81 (0.38-8.64)
	Describe any methods used to synthesize results and provide a rationale for the choice(s). If meta-analysis was performed, describe the model(s), method(s) to identify the presence and extent of statistical heterogeneity, and software package(s) used.	13d	9 (100%)	13 (59%)	13.37 (0.69-260.13)
	Describe any methods used to explore possible causes of heterogeneity among study results (e.g., subgroup analysis, metaregression).	13e	7 (78%)	14 (64%)	2.00 (0.33-12.05)
	Describe any sensitivity analyses conducted to assess the robustness of the synthesized results.	13f	4 (44%)	13 (59%)	0.55 (0.12-2.65)
Reporting bias assessment	Describe any methods used to assess the risk of bias due to missing results in a synthesis (arising from reporting biases).	14	8 (89%)	16 (73%)	3.00 (0.31-29.35)
Certainty assessment	Describe any methods used to assess certainty (or confidence) in the body of evidence for an outcome.	15	6 (67%)	9 (41%)	2.89 (0.57-14.68)
Results					
Study selection	Describe the results of the search and selection process, from the number of records identified in the search to the number of studies included in the review, ideally using a flow diagram.	16a	9 (100%)	22 (100%)	0.42 (0.01-22.96)
	Cite studies that might appear to meet the inclusion criteria, but which were excluded, and explain why they were excluded.	16b	5 (56%)	8 (36%)	2.19 (0.45-10.58)
Study characteristics	Cite each included study and present its characteristics.	17	9 (100%)	21 (95%)	1.33 (0.05-35.74)
Risk of bias in studies	Present assessments of risk of bias for each included study.	18	9 (100%)	18 (82%)	4.62 (0.22-95.60)
Results of individual studies	For all outcomes, present, for each study: (a) summary statistics for each group (where appropriate) and (b) an effect estimate and its precision (e.g. confidence/credible interval), ideally using structured tables or plots.	19	9 (100%)	22 (100%)	0.42 (0.01-22.96)
Results of syntheses	For each synthesis, briefly summarize the characteristics and risk of bias among contributing studies.	20a	4 (44%)	9 (41%)	1.16 (0.24-5.53)
	Present results of all statistical syntheses conducted. If meta-analysis was done, present for each the summary estimate and its precision (e.g. confidence/credible interval) and measures of statistical heterogeneity. If comparing groups, describe the direction of the effect.	20b	9 (100%)	22 (100%)	0.42 (0.01-22.96)
	Present results of all investigations of possible causes of heterogeneity among study results.	20c	7 (78%)	18 (82%)	0.78 (0.12-5.25)
	Present results of all sensitivity analyses conducted to assess the robustness of the synthesized results.	20d	3 (33%)	16 (73%)	0.19 (0.04-1.00)
Reporting biases	Present assessments of risk of bias due to missing results (arising from reporting biases) for each synthesis assessed.	21	7 (78%)	13 (62%)*	2.15 (0.36-13.05)
Certainty of evidence	Present assessments of certainty (or confidence) in the body of evidence for each outcome assessed.	22	6 (67%)	8 (36%)	3.50 (0.68-17.97)
Discussion					
Discussion	Provide a general interpretation of the results in the context of other evidence.	23a	9 (100%)	22 (100%)	0.42 (0.01-22.96)
	Discuss any limitations of the evidence included in the review.	23b	9 (100%)	22 (100%)	0.42 (0.01-22.96)
	Discuss any limitations of the review processes used.	23c	8 (89%)	18 (82%)	1.78 (0.177-18.53)
	Discuss the implications of the results for practice, policy, and future research.	23d	9 (100%)	22 (100%)	0.42 (0.01-22.96)
Other information					
Registration and protocol	Provide registration information for the review, including the register name and registration number, or state that the review was not registered.	24a	8 (89%)	15 (68%)	3.73 (0.39-35.93)
	Indicate where the review protocol can be accessed, or state that a protocol was not prepared.	24b	3 (33%)	7 (32%)	1.07 (0.21-5.58)
	Describe and explain any amendments to information provided at registration or in the protocol.	24c	7 (100%)*	12 (100%)*	0.60 (0.01-33.53)
Support	Describe sources of financial or non-financial support for the review, and the role of the funders or sponsors in the review.	25	6 (67%)	18 (82%)	0.44 (0.08-2.58)
Competing interests	Declare any competing interests of review authors.	26	9 (100%)	22 (100%)	0.42 (0.01-22.96)
Availability of data, code, and other materials	Report which of the following are publicly available and where they can be found: template data collection forms; data extracted from included studies; data used for all analyses; analytic code; any other materials used in the review.	27	8 (89%)	8 (36%)	14.00 (1.47-133.24)

For the group not to use the PRISMA 2020, the PRISMA 2020 adherence rate was examined according to its publication date. The number of SRs published before the base date was 66, whereas the number of SRs published after the base date was 34. The PRISMA 2020 adherence rate was 66.3% (1764/2660) for the pre-base date group and 72.3% (994/1374) for the post-base date group. The PRISMA 2020 for Abstracts adherence rate was 43.3% (343/792) for the pre-base date group and 48.5% (198/408) for the post-base date group.

**Figure 2:**
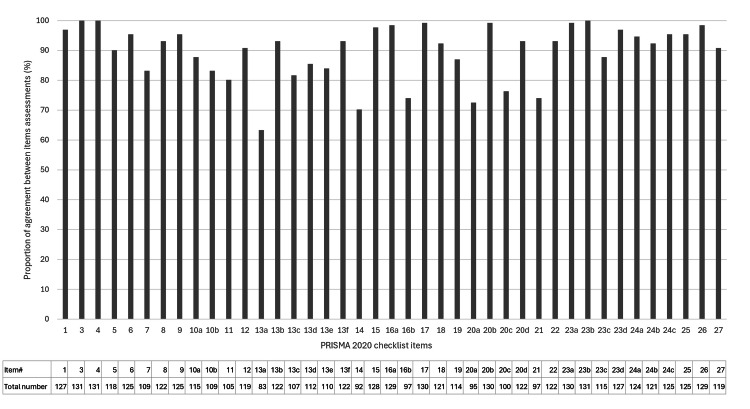
Bar chart of the proportion of agreement between the assessment by the first author and other reviewers for each item in PRISMA 2020 Bars indicate the percentage of agreement. PRISMA: Preferred Reporting Items for Systematic Reviews and Meta-Analyses The image was created by the first author, Dr. Suda.

Figure 2 shows the percentage of agreement between the assessment by the first author and other reviewers’ evaluations of each item in the PRISMA 2020 statement. The mean agreement rate for all items was 89.6% (4815/5371), with 63.4% (26/41) of the items being above average. The lowest agreement rate was for item #13a (Synthesis methods; Describe the processes used to decide which studies were eligible for each synthesis (e.g., tabulating the study intervention characteristics and comparing against the planned groups for each synthesis)), 63.4% (83/131).

**Figure 3:**
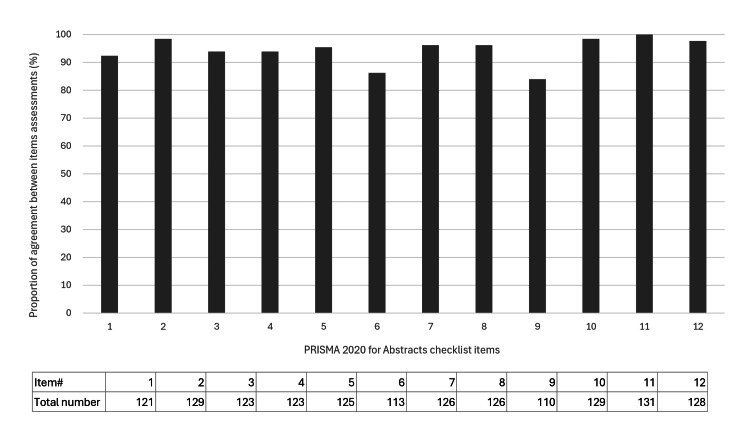
Bar chart of the proportion of agreement between the assessment by the first author assessment and the other reviewers for each item in the PRISMA 2020 for Abstracts Bars indicate the percentage of agreement. PRISMA: Preferred Reporting Items for Systematic Reviews and Meta-Analyses The image was created by the first author, Dr. Suda.

**Figure 4:**
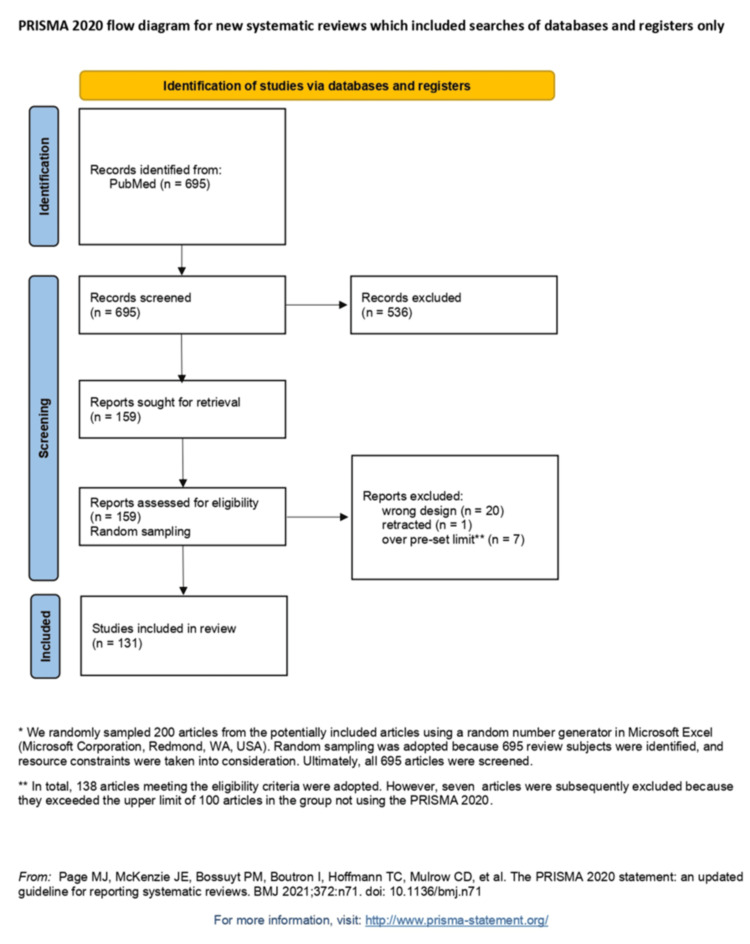
PRISMA 2020 flow diagram

**Figure 5:**
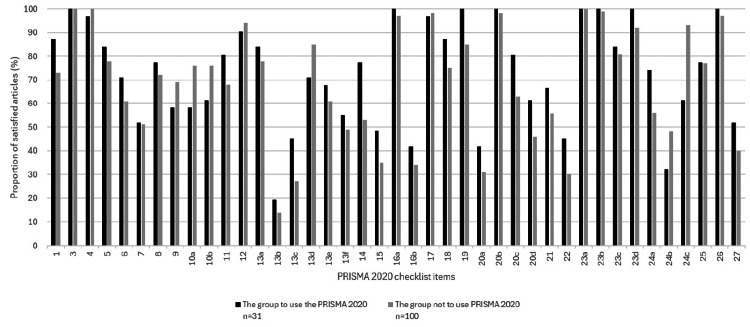
Bar chart of the adherence rate to each item of the PRISMA 2020 Black bars indicate the use of the PRISMA 2020 statement. Gray bars indicate no use of the PRISMA 2020 statement. PRISMA: Preferred Reporting Items for Systematic Reviews and Meta-Analyses The image was created by the first author, Dr. Suda.

**Figure 6:**
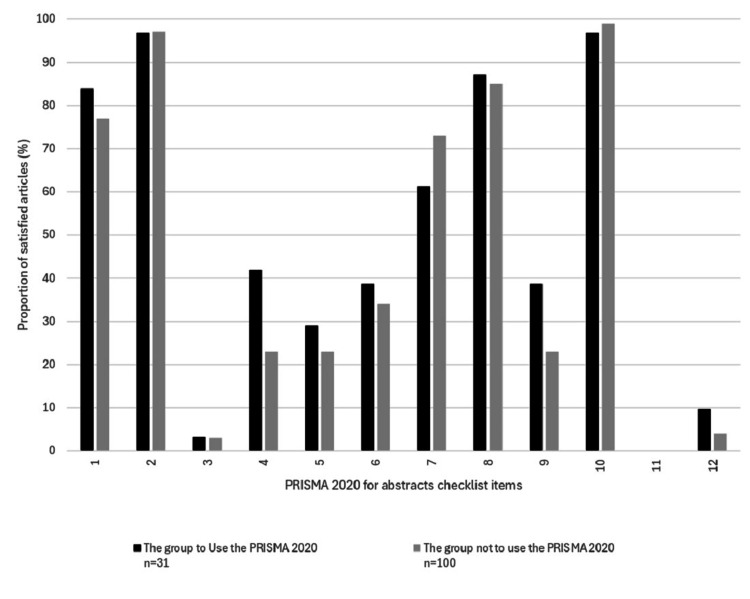
Bar chart of the adherence rate to each item of the PRISMA 2020 for Abstracts Black bars indicate the use of the PRISMA 2020 for Abstracts. Gray bars indicate no use of the PRISMA 2020 for Abstracts. PRISMA: Preferred Reporting Items for Systematic Reviews and Meta-Analyses The image was created by the first author, Dr. Suda.

The mean agreement across all items in the PRISMA 2020 for Abstracts was 94.4% (1484/1572), with 58.3% (7/12) of the items exceeding the mean (Figure 3). The lowest agreement rate was for item #9 (Data collection process; Specify the methods used to collect data from reports, including how many reviewers collected data from each report, whether they worked independently, any processes for obtaining or confirming data from study investigators, and if applicable, details of automation tools used in the process.), 84.0% (110/131).

## Discussion

This study examined the reporting quality of SRs in emergency medicine journals using the PRISMA 2020 statement. The results showed that overall adherence rates improved with using PRISMA 2020. We also showed that the PRISMA 2020 for Abstracts did not lead to significant improvements, compared with SRs that did not use the PRISMA 2020 statement. Although we anticipated that the updated PRISMA 2020 guidelines would elevate the reporting standards in emergency medicine journals, the findings of this study indicate that the impact was inadequate.

The low number of papers using PRISMA 2020, even though it was updated in 2020. That may be because of insufficient awareness of authors, lack of thorough PRISMA 2020 recommendations in some journals, and insufficient guidance from editors and reviewers. Past studies have shown that reviews published in journals recommending or requiring adherence to a reporting guideline had better reporting quality than journals without such instructions [23].

The mean overall adherence to PRISMA 2020 among our sample was 69.4% (3683/5304), which is higher than the previous report that examined PRISMA 2020 adherence rates in radiology [8]. These results are similar to those of earlier reports on PRISMA 2009 in other fields that reported adherence rates of 61.4-72.2% [10-12,15], adherence to 19-20 of 27 items [16,18]. The section with the highest adherence rate was the introduction section, consistent with a previous report. The lowest adherence rate was found in the methods section, which differs from a previous study [8]. Of the 41 items (excluding item #2) in the PRISMA 2020 statement, 23 in the group to use the PRISMA 2020 and 27 in the group not to use the PRISMA 2020 were reported in fewer than 80% of the articles. This result is similar to previous literature [8]. Previous literature on PRISMA 2020 in the radiology field [8] and PRISMA 2009 in the emergency medicine field [23] has highlighted concerns about inadequate reporting quality. None of the reviews adequately reported on all items, a result similar to the radiology SR for PRISMA 2020 [8] and the emergency SR for PRISMA 2009 [23].

Implementing the PRISMA 2020 statement enhanced the reporting quality. However, only one of the 41 items improved significantly in terms of reporting quality for each item. Item #13c (Synthesis methods; Describe any methods used to tabulate or visually display results of individual studies and syntheses.), #15 (Certainty assessment; Describe any methods used to assess certainty (or confidence) in the body of evidence for an outcome), #16b (Study selection; Cite studies that might appear to meet the inclusion criteria, but which were excluded, and explain why they were excluded.), #20a (Results of syntheses; For each synthesis, briefly summarize the characteristics and risk of bias among contributing studies.), #22 (Certainty of evidence; Present assessments of certainty (or confidence) in the body of evidence for each outcome assessed.), and 24b (Registration and protocol; Indicate where the review protocol can be accessed, or state that a protocol was not prepared.) had lower reporting rates, even in the group to use the PRISMA 2020. This result is similar to previous literature [24].

The mean overall adherence to PRISMA 2020 for Abstracts among our sample was 46.0% (723/1572), which is slightly higher than the previous report in the field of radiology [8]. Item #4 (Information sources; Specify the information sources (e.g. databases, registers) used to identify studies and the date when each was last searched.), #5 (Risk of bias; Specify the methods used to assess the risk of bias in the included studies.), #6 (Synthesis of results; Specify the methods used to present and synthesize results.), #9 (Limitations of evidence; Provide a brief summary of the limitations of the evidence included in the review (e.g. study risk of bias, inconsistency, and imprecision).), #11 (Funding; Specify the primary source of funding for the review.), and #12 (Registration; Provide the register name and registration number.) had lower reporting rates, even in the group to use the PRISMA 2020. One possible reason for the lack of significant improvement in adherence rates in articles using PRISMA 2020 may be that researchers and their supervisors do not fully understand the guidelines. In addition, PRISMA 2020 is detailed and contains many items that may be a barrier to effective adherence.

In the group to use the PRISMA 2020, only nine (29%) attached the checklist. The PRISMA 2020 adherence rate was examined for the group to use the PRISMA 2020 with and without the attached checklist, but only item #27 showed significant improvement with the attached checklist. We considered many papers that used the PRISMA 2020 checklist but did not attach it to their references.

The adherence rate was higher in the literature published after March 29, 2022, one year after the update of PRISMA 2020. Although we expected the reporting quality would be lower in the literature that did not mention using PRISMA 2020, even after the announcement and dissemination of the updated statement, the actual results were different. We considered the possibility of cases in which the authors did not mention using PRISMA 2020 in their papers but used it to prepare their papers. In the endoscopic ultrasound diagnosis field, SR and meta-analyses reporting and methodology quality have markedly improved since the PRISMA and AMSTAR (A Measurement Tool to Assess Systematic Reviews) checklists were published [25].

Simply stating the use of the PRISMA 2020 statement is inadequate for enhancing the reporting quality in SRs. Thus, we recommend that authors not only declare their use of the PRISMA 2020 guidelines but also accurately adhere to each item. Several years after the publication of PRISMA 2020, the quality of reporting remains low despite the checklist and its complementary guidelines. Further recommendations, additional expert support, educational programs for researchers and reviewers, policy reviews by individual journals, or new approaches are needed.

This review possesses several strengths. First, it is a pioneering study that evaluates the impact of the PRISMA 2020 statement on the reporting quality of SRs in the emergency medicine field. Our findings bolster the current understanding of the weaknesses in SRs within emergency medicine and highlight the necessity for strategies to enhance the quality of these reviews. Second, to minimize the risk of measurement error, two authors independently selected studies and extracted data.

However, this study has limitations. Although we planned to examine 100 SRs in the group to use the PRISMA 2020, only 31 met the criteria, resulting in a small sample. Selection bias was present owing to the inclusion of only English-language articles, the restriction to a single database, and the focus on the field of emergency medicine. Consequently, the findings of this study cannot be generalized to SRs published in other languages, databases, or disciplines. In addition, the country of the first author was evaluated based on previous research. The country of the corresponding author and last author were not evaluated. When a last author or corresponding author from a different country plays a leading role in a study, there is a risk that the influence of that country may be overlooked, and regional and cultural characteristics may not be reflected.

## Conclusions

This study demonstrated that implementing the PRISMA 2020 improved the reporting quality of SRs in emergency medicine journals. However, in terms of reporting quality for each item, only one of the 41 items improved significantly. For further improvement, merely declaring the use of the PRISMA 2020 guidelines is insufficient. Researchers must adhere to each item of the statement when conducting SRs in the emergency medicine field.

## Appendices

**Table 5:** Included emergency medicine journal lists

Journal name	Journal Citation Reports Abbreviation	Journal Search term in MEDLINE (PubMed)
Emergency	EMERGENCY	"Emergency"[jour]
Emergency Radiology	EMERG RADIOL	"Emerg Radiol"[jour]
Emergency Medicine Journal	EMERG MED J	"Emerg Med J"[jour]
Emergency Medicine Australasia	EMERG MED AUSTRALAS	"Emerg Med Australas"[jour]
Emergency Medicine International	EMERG MED INT	"Emerg Med Int"[jour]
Emergency Medicine Clinics of North America	EMERG MED CLIN N AM	"Emerg Med Clin North Am"[jour]
BMC Emergency Medicine	BMC EMERG MED	"BMC Emerg Med"[jour]
Current Emergency and Hospital Medicine Reports	CURR EMERG HOSP ME R	"Curr Emerg Hosp Med Rep"[jour]
Academic Emergency Medicine	ACAD EMERG MED	"Acad Emerg Med"[jour]
Advanced Emergency Nursing Journal	ADV EMERG NURS J	"Adv Emerg Nurs J"[jour]
Pediatric Emergency Care	PEDIATR EMERG CARE	"Pediatr Emerg Care"[jour]
Annals of Emergency Medicine	ANN EMERG MED	"Ann Emerg Med"[jour]
Journal of Emergency Nursing	J EMERG NURS	"J Emerg Nurs"[jour]
Journal of Emergency Medicine	J EMERG MED	"J Emerg Med"[jour]
Prehospital Emergency Care	PREHOSP EMERG CARE	"Prehosp Emerg Care"[jour]
Open Access Emergency Medicine	OPEN ACCESS EMERG M	"Open Access Emerg Med"[jour]
Australasian Emergency Care	AUSTRALAS EMERG CARE	"Australas Emerg Care"[jour]
Internal and Emergency Medicine	INTERN EMERG MED	"Intern Emerg Med"[jour]
International Emergency Nursing	INT EMERG NURS	"Int Emerg Nurs"[jour]
World Journal of Emergency Surgery	WORLD J EMERG SURG	"World J Emerg Surg"[jour]
World Journal of Emergency Medicine	WORLD J EMERG MED	"World J Emerg Med"[jour]
African Journal of Emergency Medicine	AFR J EMERG MED	"Afr J Emerg Med"[jour]
Turkish Journal of Emergency Medicine	TURK J EMERG MED	"Turk J Emerg Med"[jour]
Western Journal of Emergency Medicine	WEST J EMERG MED	"West J Emerg Med"[jour]
American Journal of Emergency Medicine	AM J EMERG MED	"Am J Emerg Med"[jour]
Advanced Journal of Emergency Medicine	ADV J EMERG MED	"Adv J Emerg Med"[jour]
Canadian Journal of Emergency Medicine	CAN J EMERG MED	"CJEM"[jour]
European Journal of Emergency Medicine	EUR J EMERG MED	"Eur J Emerg Med"[jour]
Archives of Academic Emergency Medicine	ARCH ACAD EMERG MED	"Arch Acad Emerg Med"[jour]
Journal of Accident & Emergency Medicine	J ACCID EMERG MED	"J Accid Emerg Med"[jour]
International Journal of Emergency Medicine	INT J EMERG MED	"Int J Emerg Med"[jour]
Clinical and Experimental Emergency Medicine	CLIN EXP EMERG MED	"Clin Exp Emerg Med"[jour]
European Journal of Trauma and Emergency Surgery	EUR J TRAUMA EMERG S	"Eur J Trauma Emerg Surg"[jour]
Journal of Homeland Security and Emergency Management	J HOMEL SECUR EMERG	"J Homel Secur Emerg Manag"[jour]
Journal of the American College of Emergency Physicians Open	JACEP OPEN	"J Am Coll Emerg Physicians Open"[jour]
Scandinavian Journal of Trauma Resuscitation & Emergency Medicine	SCAND J TRAUMA RESUS	"Scand J Trauma Resusc Emerg Med"[jour]
Ulusal Travma ve Acil Cerrahi Dergisi-Turkish Journal of Trauma & Emergency Surgery	ULUS TRAVMA ACIL CER	"Ulus Travma Acil Cerrahi Derg"[jour]

**Table 6:** Search strategies using MEDLINE (PubMed)

Search strategies
#1 2021[pdat] OR 2022[pdat] OR 2023[pdat]
#2 "Emergency"[jour] OR "Emerg Radiol"[jour] OR "Emerg Med J"[jour] OR "Emerg Med Australas"[jour] OR "Emerg Med Int"[jour] OR "Emerg Med Clin North Am"[jour] OR "BMC Emerg Med"[jour] OR "Curr Emerg Hosp Med Rep"[jour] OR "Acad Emerg Med"[jour] OR "Adv Emerg Nurs J"[jour] OR "Pediatr Emerg Care"[jour] OR "Ann Emerg Med"[jour] OR "J Emerg Nurs"[jour] OR "J Emerg Med"[jour] OR "Prehosp Emerg Care"[jour] OR "Open Access Emerg Med"[jour] OR "Australas Emerg Care"[jour] OR "Intern Emerg Med"[jour] OR "Int Emerg Nurs"[jour] OR "World J Emerg Surg"[jour] OR "World J Emerg Med"[jour] OR "Afr J Emerg Med"[jour] OR "Turk J Emerg Med"[jour] OR "West J Emerg Med"[jour] OR "Am J Emerg Med"[jour] OR "Adv J Emerg Med"[jour] OR "CJEM"[jour] OR "Eur J Emerg Med"[jour] OR "Arch Acad Emerg Med"[jour] OR "J Accid Emerg Med"[jour] OR "Int J Emerg Med"[jour] OR "Clin Exp Emerg Med"[jour] OR "Eur J Trauma Emerg Surg"[jour] OR "J Homel Secur Emerg Manag"[jour] OR "J Am Coll Emerg Physicians Open"[jour] OR "Scand J Trauma Resusc Emerg Med"[jour] OR "Ulus Travma Acil Cerrahi Derg"[jour]
#3 (("Meta-Analysis as Topic"[MeSH] OR meta analy*[TIAB] OR metaanaly*[TIAB] OR "Meta-Analysis"[PT] OR "Systematic Review"[PT] OR "Systematic Reviews as Topic"[MeSH] OR systematic review*[TIAB] OR systematic overview*[TIAB] OR "Review Literature as Topic"[MeSH]) OR (cochrane[TIAB] OR embase[TIAB] OR psychlit[TIAB] OR psyclit[TIAB] OR psychinfo[TIAB] OR psycinfo[TIAB] OR cinahl[TIAB] OR cinhal[TIAB] OR "science citation index"[TIAB] OR bids[TIAB] OR cancerlit[TIAB]) OR (reference list*[TIAB] OR bibliograph*[TIAB] OR hand-search*[TIAB] OR "relevant journals"[TIAB] OR manual search*[TIAB]) OR (("selection criteria"[TIAB] OR "data extraction"[TIAB]) AND "Review"[PT])) NOT ("Comment"[PT] OR "Letter"[PT] OR "Editorial"[PT] OR ("Animals"[MeSH] NOT ("Animals"[MeSH] AND "Humans"[MeSH])))
#4 “individual patient data”[TIAB] OR "Network Meta-Analysis"[Mesh] OR “network meta*” [TIAB] OR “scoping review*” [TIAB] OR “mapping review*”[TIAB] OR “scoping study”[TIAB] OR “diagnostic test”[TIAB]
#5 #1 AND #2 AND #3 NOT #4

**Table 7:** Excluded journal lists Not listed in MEDLINE (PubMed)

Emergency Care Journal, Journal of Emergency Medicine Case Reports, Frontiers in Emergency Medicine, Clinical Pediatric Emergency Medicine, Eurasian Journal of Emergency Medicine, Hong Kong Journal of Emergency Medicine, Australian Journal of Emergency Management, Journal of Veterinary Emergency and Critical Care, International Journal of Emergency Services, International Journal of Emergency Management
